# Post-partum myocardial ischemia due to intramuscular methylergonovine-induced coronary vasospasm: case report

**DOI:** 10.1186/s12872-023-03216-9

**Published:** 2023-04-17

**Authors:** Sae K Jang, Kathryn Berlacher, Alisse Hauspurg

**Affiliations:** 1 Department of Cardiology, University of Pittsburgh Medical Center, 200 Lothrop Street S-553 Scaife Hall, Pittsburgh, PA 15213 USA; 2 Department of Obstetrics, Gynecology and Reproductive Sciences, University of Pittsburgh Medical Center, Pittsburgh, USA

**Keywords:** Postpartum methylergonovine, Coronary vasospasm, Case report

## Abstract

**Background:**

Methylergonovine is a vasoconstrictive agent historically used as a provocative agent in the lab for coronary vasospasm; it is also a first line uterotonic agent for management of postpartum hemorrhage.

**Case Presentation:**

A 29-year-old female with history of smoking and idiopathic thrombocytopenia received intramuscular methylergonovine after delivery of twins for intrauterine hemorrhage management. Subsequently, she had episodes of chest pain with high sensitivity Troponin I elevation to 1509 ng/L with accompanying septal T wave inversions, decreased left ventricular ejection fraction to 49% and basal septal wall hypokinesis. Computed tomography (CT) coronary angiogram showed patent coronary arteries and no coronary arterial dissection. The patient was conservatively managed with aspirin and metoprolol, and on follow up had fully recovered left ventricular function with resolution of wall motion abnormalities. Given this, coronary vasospasm due to intramuscular methylergonovine is the most likely cause of patient’s chest pain and associated myocardial ischemia.

**Conclusions:**

Intramuscular, intrauterine, intravenous, and even oral methylergonovine can rarely cause coronary vasospasm leading to myocardial ischemia. Cardiologists caring for postpartum patients should be aware of these potential lethal complications; prompt identification and administration of sublingual nitroglycerin can prevent severe complications of arrythmias, heart block, or cardiac arrest.

**Supplementary Information:**

The online version contains supplementary material available at 10.1186/s12872-023-03216-9.

## Case presentation

**Fig. 1:**
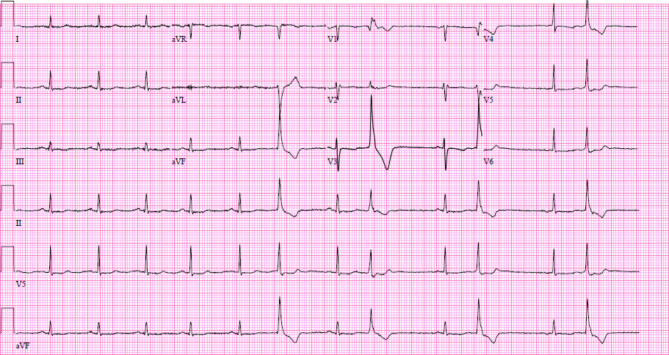
Initial electrocardiogram obtained at the time of chest pain showing T wave flattening in inferior and anterolateral leads, as well as premature ventricular contractions

A 29-year-old female with past medical history of one pack-year smoking and mild idiopathic thrombocytopenia was admitted for preterm premature rupture of membranes with a dichorionic twin pregnancy at 34 weeks of gestation. Her presenting hemoglobin was 10.2 g/dL and platelet count was 84/nL. Labor was induced with oxytocin and epidural anesthesia was used. Her history is notable for a COVID-19 infection at 30 weeks; otherwise pregnancy was unremarkable. She delivered twins via vaginal delivery with a breech extraction of twin B. Due to postpartum hemorrhage, she received uterine massage and exploration. Her fundus was noted to be firm with continued bleeding thought to be from lower uterine segment atony. Her oxytocin dose was doubled in dose, and ultimately, she received two doses of intramuscular methylergonovine 0.2 mg (standard obstetric dosing). Immediately after returning from the operating room, the patient reported chest tightness with radiation to her neck, and associated shortness of breath. Telemetry showed intermittent sinus bradycardia in the 20–30’s, and frequent atrial ectopy. Initial electrocardiogram (ECG) (Fig. 1) showed T wave flattening in inferior and anterolateral leads, as well as premature ventricular contractions. Her chest pain resolved spontaneously within a few minutes.

The differential diagnosis for the patient’s chest pain included spontaneous coronary artery dissection (SCAD), coronary vasospasm, acute coronary plaque rupture, and pulmonary embolism.

**Fig. 2:**
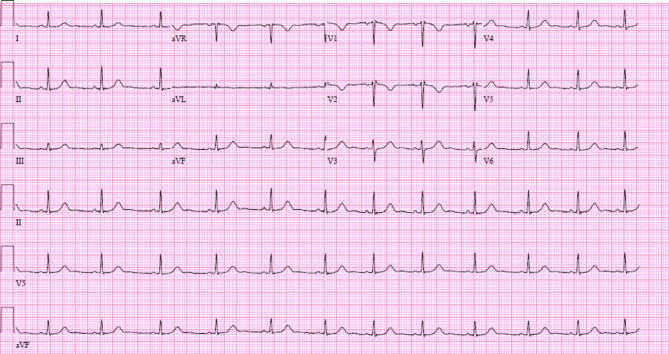
Repeat ECG at time of second episode of chest pain showed T wave inversions in septal leads

CT angiography (CTA) of the chest was negative for pulmonary embolism. Laboratory testing showed an initial normal-range high sensitivity troponin I (hsTnI) at 20 ng/L. Approximately eight hours later, the patient had another brief episode of chest pain. Nitroglycerin was not given as chest pain resolved in minutes without intervention. Recheck of hsTnI showed an uptrend to a peak of 1509 ng/L. Hemoglobin remained stable at 10 g/dL. Repeat ECG showed T wave inversions in septal leads (Fig. 2).

Transthoracic echocardiogram showed left ventricular ejection fraction of 49% with basal septal wall hypokinesis (Supplemental Videos 1–3).

Given the rapid resolution of her chest pain, the patient received one dose of 325 mg of aspirin. Heparin drip was deferred due to concern for potential SCAD and further propagation of coronary dissection.

Coronary angiography was discussed with the patient, but ultimately due to lower suspicion of a coronary occlusive event, a dedicated CT coronary angiogram was obtained, which showed patent coronary arteries without obstruction or dissection.

She was started on metoprolol succinate 25 mg daily for mildly reduced ejection fraction and was discharged with cardiology follow up.

On three-month outpatient cardiology follow up, a repeat transthoracic echocardiogram showed normal ejection fraction of 60–65% with no wall motion abnormality. Patient had no further episodes of chest pain. Review of the event with patient and her family elucidated the administration of methylergonovine just prior to her chest pain episode. Given normalization of cardiac function, transient chest pain right after intramuscular methylergonovine, coronary vasospasm was felt to be the most likely cause of myocardial ischemia. Metoprolol was discontinued and amlodipine 5 mg daily was added for vasospasm prevention.

## Discussion

Methylergonovine is an ergot alkaloid that induces contractions of uterine and vascular smooth muscles. It is the first-line agent used for management of postpartum hemorrhage. Previously, intravenous methylergonovine was used as a provocative agent in the coronary angiography laboratories to augment diagnosis of coronary vasospasm. However, reported complications of methylergonovine in coronary angiography suites included ventricular fibrillation, ventricular tachycardia, third-degree atrioventricular block, and sinus bradycardia, with a reported complication rate of 5.2% [1–3]. Intracoronary methylergonovine is no longer used in the catheterization laboratory.

To date, only a handful of case reports of myocardial infarction related to obstetric use of methylergonovine have been reported [4–7]. To our knowledge, this is the first reported case of coronary vasospasm due to intramuscular methylergonovine use. Though rare, complications from obstetric use of methylergonovine include myocardial infarction (confirmed at autopsy), cardiac arrest, and death [4, 5]. There is not enough data to assess whether uterine versus intramuscular injection is safer.

In this patient, chest pain was accompanied by hsTnI elevation, septal hypokinesis on echocardiogram, and a normal coronary CTA, suggesting coronary vasospasm as cause for supply-demand mismatch in septal perforator. There were also T wave inversions in V1 and V2, which could correlate to the septal distribution of myocardial ischemia, though there was no baseline ECG prior to this encounter to compare; T wave inversions in anteroseptal leads could also be seen with varying placement of precordial leads. Given the rarity of significant vasospasm from postpartum use of methylergonovine, it is likely that the patient has an unidentified predisposition to vasospasm. Thus, we elected to treat with a dihydropyridine calcium channel blocker for prevention.

## Conclusions

Intramuscular, intrauterine, intravenous, and even oral methylergnovine [8] can rarely cause coronary vasospasm leading to myocardial ischemia. Given that this is a first-line agent for management of obstetric hemorrhage, cardiologists caring for postpartum patients should be aware of these potential lethal complications, especially as the field of cardio-obstetrics continues to grow. Reported adverse outcomes in literature also include myocardial infarction, cardiac arrest, and death. Prompt identification and administration of sublingual nitroglycerin can prevent severe complications of arrythmias, heart block, or cardiac arrest.

## Electronic supplementary material

Below is the link to the electronic supplementary material.

Supplementary Material 1

Supplementary Material 2

Supplementary Material 3

Supplementary Material 4

## Data Availability

All data generated or analyzed during this study are included in this published article and/or are available from the corresponding author on reasonable request.
