# Supplementary material for: Post-partum myocardial ischemia due to intramuscular methylergonovine-induced coronary vasospasm: case report
Source: BMC Cardiovasc Disord. 2023 Apr 17;23:199. doi: 10.1186/s12872-023-03216-9 (PMC10109221; doi:10.1186/s12872-023-03216-9)
Supplement: Supplementary file 4 — Supplementary Material 4 [file 12872_2023_3216_MOESM4_ESM.docx]

**Supplemental Materials**

**Supplemental Videos 1-3:** Transthoracic echocardiogram showed basal septal wall hypokinesis and mildly reduced ejection fraction.
